# Optical mapping and optogenetics in cardiac electrophysiology research and therapy: a state-of-the-art review

**DOI:** 10.1093/europace/euae017

**Published:** 2024-01-16

**Authors:** Olivia Baines, Rina Sha, Manish Kalla, Andrew P Holmes, Igor R Efimov, Davor Pavlovic, Christopher O’Shea

**Affiliations:** Institute of Cardiovascular Sciences, College of Medical and Dental Science, University of Birmingham, Edgbastion, Wolfson Drive, Birmingham B15 2TT, UK; Institute of Cardiovascular Sciences, College of Medical and Dental Science, University of Birmingham, Edgbastion, Wolfson Drive, Birmingham B15 2TT, UK; Institute of Cardiovascular Sciences, College of Medical and Dental Science, University of Birmingham, Edgbastion, Wolfson Drive, Birmingham B15 2TT, UK; Institute of Cardiovascular Sciences, College of Medical and Dental Science, University of Birmingham, Edgbastion, Wolfson Drive, Birmingham B15 2TT, UK; Department of Biomedical Engineering, Northwestern University, Evanston, IL, USA; Department of Medicine, Division of Cardiology, Northwestern University, Evanston, IL, USA; Institute of Cardiovascular Sciences, College of Medical and Dental Science, University of Birmingham, Edgbastion, Wolfson Drive, Birmingham B15 2TT, UK; Institute of Cardiovascular Sciences, College of Medical and Dental Science, University of Birmingham, Edgbastion, Wolfson Drive, Birmingham B15 2TT, UK

**Keywords:** Optogenetic, Optical mapping, All-optical, Optoelectronic, Fluorescence, Cardiac, Action potential, Calcium, Pacemaker

## Abstract

State-of-the-art innovations in optical cardiac electrophysiology are significantly enhancing cardiac research. A potential leap into patient care is now on the horizon. Optical mapping, using fluorescent probes and high-speed cameras, offers detailed insights into cardiac activity and arrhythmias by analysing electrical signals, calcium dynamics, and metabolism. Optogenetics utilizes light-sensitive ion channels and pumps to realize contactless, cell-selective cardiac actuation for modelling arrhythmia, restoring sinus rhythm, and probing complex cell–cell interactions. The merging of optogenetics and optical mapping techniques for ‘all-optical’ electrophysiology marks a significant step forward. This combination allows for the contactless actuation and sensing of cardiac electrophysiology, offering unprecedented spatial–temporal resolution and control. Recent studies have performed all-optical imaging *ex vivo* and achieved reliable optogenetic pacing *in vivo*, narrowing the gap for clinical use. Progress in optical electrophysiology continues at pace. Advances in motion tracking methods are removing the necessity of motion uncoupling, a key limitation of optical mapping. Innovations in optoelectronics, including miniaturized, biocompatible illumination and circuitry, are enabling the creation of implantable cardiac pacemakers and defibrillators with optoelectrical closed-loop systems. Computational modelling and machine learning are emerging as pivotal tools in enhancing optical techniques, offering new avenues for analysing complex data and optimizing therapeutic strategies. However, key challenges remain including opsin delivery, real-time data processing, longevity, and chronic effects of optoelectronic devices. This review provides a comprehensive overview of recent advances in optical mapping and optogenetics and outlines the promising future of optics in reshaping cardiac electrophysiology and therapeutic strategies.

## Graphical Abstract

**Graphical Abstract:**
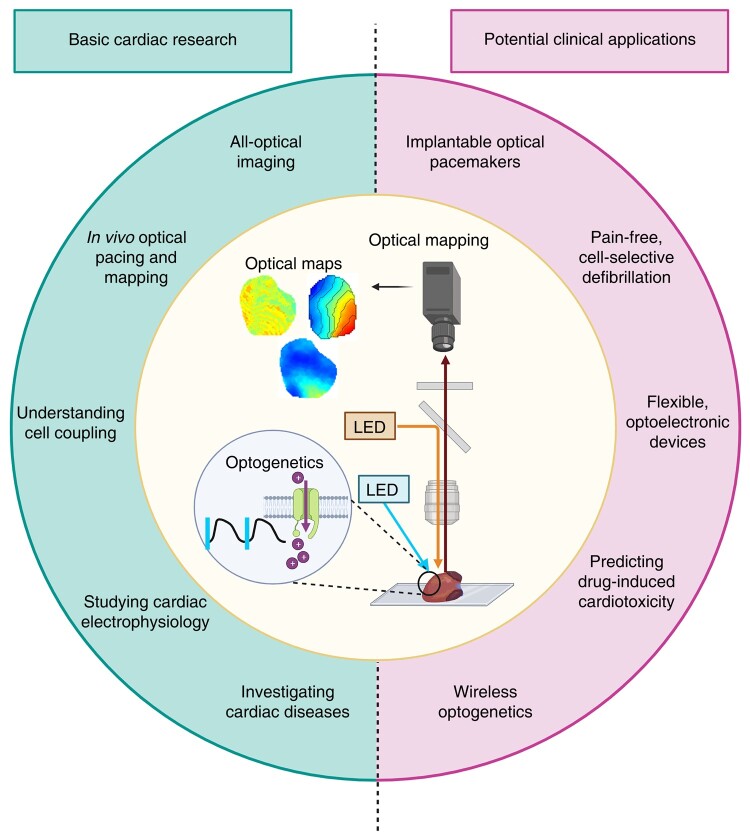

**What’s new?**

- Recent technical breakthroughs in optical mapping and optogenetics are revolutionising cardiac research.
- Advancements include new motion tracking approaches, fluorescent probes and opsins offering new insights into cardiac electrophysiology.
- The integration of optogenetics and optical mapping into an ‘all-optical’ approach offers contactless actuation and sensing of cardiac electrophysiology, providing unprecedented spatial-temporal resolution and control.
- Developments in optoelectronics are enabling the creation of miniaturised, biocompatible implantable cardiac devices like pacemakers and defibrillators with optoelectrical closed-loop systems.
- Application of optical methods to patient care are on the horizon, however challenges remain such as opsin delivery, real-time data processing, device longevity, and understanding the chronic effects of optoelectronic devices.

## Introduction

**Figure 1:**
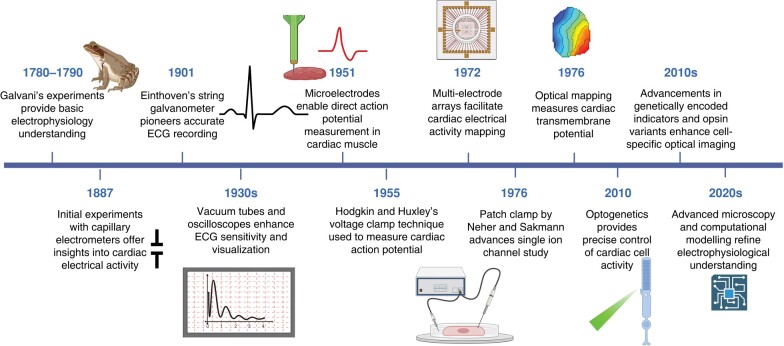
Historical timeline of the developments of novel cardiac electrophysiology tools and their utility. ECG, electrocardiogram. Figure created on BioRender.com.

Optical approaches for studying electrical function in the heart have fundamentally shaped our understanding of cardiac electrophysiology for 50 years (*Figure 1*).^1^ The origins of electrophysiology can be traced back to Galvani’s experiments in the 18th century, demonstrating intrinsic electrical activity generates muscle contraction in frog legs.^2^ Development of the capillary electrometer then allowed first recordings of cardiac electrical activity, leading to Einthoven’s refinement of the electrocardiogram (ECG).^3^ Microelectrode recordings from Purkinje fibres generated the first recorded cardiac action potentials.^4^ Subsequent single-cell techniques, such as voltage and patch-clamping, informed our understanding of distinct action potential phases and respective currents.^5^ However, the limited scalability and throughput prompted the development of multi-electrode arrays (MEAs), enabling measurement of electrical propagation.^6^ Nevertheless, MEAs only record extracellular potential and spatial resolution is constrained by electrode distance. Optical imaging overcomes these limitations in cardiac electrophysiological interrogation, offering unmatched spatiotemporal resolution.

Cardiac optical mapping uses voltage and calcium (Ca^2+^)-sensitive dye to image multi-cellular preparations at high spatiotemporal resolutions.^1^ Cultured cardiomyocytes,^7^ induced pluripotent stem cell-derived cardiomyocytes (iPSC-CMs),^8^ engineered heart tissue (EHT),^9^ myocardial slices,^10^ isolated atria,^11^ and whole hearts^12^ have all been optically mapped. It has been crucial in deciphering the role of rotors in atrial fibrillation,^13^ the virtual electrode phenomena,^14^ and autonomic regulation of cardiac arrhythmias.^15^ Optical mapping is continually advancing with novel dye variants, improved hardware, motion tracking, and analysis tools.^16^

Optogenetics uses light to actuate transmembrane ion movement to modulate cardiac excitability in tissues expressing light-sensitive proteins called opsins.^17^ Optogenetic applications include precise control of pacing^18^ and arrhythmia induction^19^ or termination.^20^ Optogenetics provides huge potential for cardiac pacemaker development and defibrillation. Unlike chemical and electrical stimulation, optogenetics utilizes contactless, cell-selective pacing with minimal cytotoxicity. More recently, optical imaging and optogenetics have been combined to realize ‘all-optical’ electrophysiology, enabling precise control and measurement of cardiac function using light alone.^21,22^

This review focuses on the applications, recent advances, and limitations of optogenetics, optical mapping and all-optical imaging systems for cardiac electrophysiology mechanistic research and translational applications.

## Principles of optical mapping

### Optical mapping fluorescent sensors and illumination

**Figure 2:**
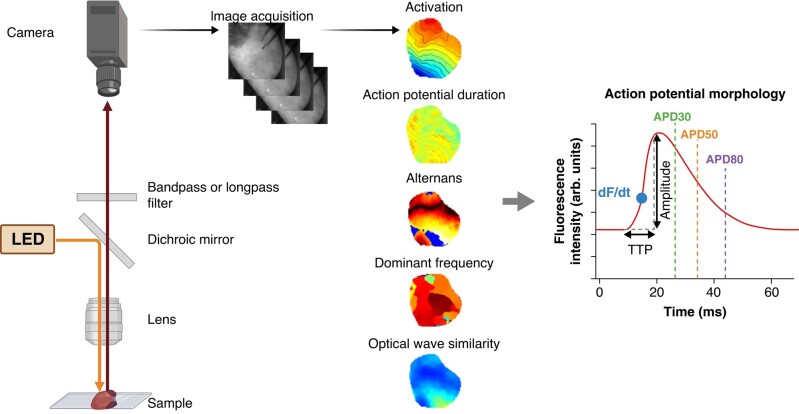
Principles of optical mapping. Typically, a fluorescent sensor absorbs photons at wavelength-specific illumination, provided by a light source (e.g. light-emitting diode), and emits lower energy photons at a longer wavelength. Crucially, the properties of the emitted spectrum (peak wavelength, brightness, etc.) are responsive to a physiological property of the cell, typically transmembrane voltage or Ca^2+^ concentration. Emitted fluorescence can be passed through a filter for wavelength-specific transmission and removal of background noise. A photodetector captures the final fluorescent signal to quantify fractional change in fluorescence typically due to voltage or Ca^2+^ changes, and optical maps are generated. Action potential morphology parameters can subsequently be calculated including action potential duration; the time between dF/dt (upstroke) and 30% (APD30), 50% (APD50), or 80% (APD80) of repolarization, for example. LED, light-emitting diode; APD, action potential duration; TTP, time-to-peak. dF/dt denotes upstroke. Figure created on BioRender.com.

Optical mapping is a fluorescence-based technique that visualizes electrophysiological properties of multi-cellular preparations at unparalleled spatiotemporal resolution (*Figure 2*). This method involves the infusion of cardiac preparations, ranging from cellular monolayers to whole hearts, with voltage and/or Ca^2+^ fluorescent sensors. Once these indicators are illuminated [e.g. by light-emitting diodes (LEDs)], the resulting fluorescence is captured by high-speed cameras.^23^ Consequently, optical recordings of cardiac action potentials, Ca^2+^ transient morphology, and conduction are obtained that give crucial electrophysiological insights in health and disease.

### Established and novel optical mapping probes

#### Voltage-sensitive dyes

The coordinated generation and propagation of cardiac action potentials form the electrical basis of the heartbeat. For this reason, synthetic voltage-sensitive dyes are the most used in optical mapping. These dyes [e.g. di-4-aminonaphthylethenylpyridinium (di-4-ANEPPS)] respond to changes in voltage in the picosecond range, enabling accurate optical recording of surface cardiac electrophysiology.^24^

Voltage dyes are crucial tools for optical mapping; however, they are not without limitation. The fractional change in fluorescence output is low, which can generate low-quality signals. Moreover, most commonly used dyes are optimally excited by blue/green light, limiting penetration depth and promoting phototoxic tissue interactions.^24^ Further, synthetic dyes can have potential adverse effects on electrophysiology. For example, voltage-sensitive dye di-4-ANEPPS has demonstrated reduced spontaneous heart rate, sodium current, T-wave amplitude, and AV-node conduction in *ex vivo* and *in vitro* preparations.^12,25^

Red-shifted dyes help overcome some of these issues. Di-4-ANBDQBS is a potentiometric dye with red excitation (660 nm) and near-infrared emission, for greater penetration depth.^26^ Di-4-ANBDQBS has been used to capture information from the endocardium^26^ with minimal effects on cardiac electrophysiology and cardiotoxicity.^23,27^

Most synthetic sensors show adequate stability for acute electrophysiology investigation but exhibit signal decay over time due to photobleaching and dye leakage. Recently developed photo-electron transfer dyes, such as fluoVolt, show high photostability, rapid response time (pico- to nanoseconds), and high fractional changes.^28^ Additionally, high sensitivity makes these dyes better suited to two-photon imaging application, allowing greater penetration depth for transmural investigation.^29^

#### Calcium probes

Ca^2+^ couples the cardiomyocyte action potential to contraction.^30^ Imaging intracellular Ca^2+^ ([Ca^2+^]_i_) for Ca^2+^ transient recording is another significant application of cardiac optical mapping, often conducted simultaneously with voltage imaging.^31^ Ca^2+^ probes typically consist of a fluorophore, chelator, and conjugator to quantify [Ca^2+^]_i_. The most common of these are rhodamine (Rhod-2)-based probes such as rhod-2-AM, used to image cytosolic [Ca^2+^]_i_.^32^

Recent advances have enabled organelle-specific [Ca^2+^]_i_ imaging. Valverde *et al.*^33^ recorded sarcoplasmic reticulum Ca^2+^ transients alongside cytosolic Ca^2+^ transients in isolated murine whole hearts using pulsed local-field fluorescence microscopy of mag-fluo-4 AM and rhod-2-AM. Trollinger *et al.*^34^ developed a novel technique to achieve mitochondrial-specific [Ca^2+^]_i_ measurement via a cold/warm rhod-2-AM loading protocol while simultaneously recording cytosolic [Ca^2+^]_i_ using fluo3.

Several Ca^2+^ indicators are also suitable for ratiometry, measuring the ratio of emission signals at different excitation wavelengths (‘excitation ratiometry’), as they exhibit wavelength-dependent fluorescence output. This enables more accurate quantification of absolute [Ca^2+^]_i_ in single-cell models and Ca^2+^ amplitudes in whole heart optical mapping.^35^ Furthermore ratiometric dyes, including voltage-sensitive dyes,^36^ can significantly mitigate system noise and motion artefacts (see Motion tracking section).^37^

#### Genetically encoded voltage and calcium sensors

Genetically encoded voltage and Ca^2+^ sensors (GEVI/GECI) can achieve durable, cell-specific expression for long-term cardiac electrophysiology *in vitro* studies with reduced cytotoxicity, offering unique capabilities compared with synthetic indicators.^38^ However, their adoption is hindered by slower response times compared with ‘fast’ synthetic dyes and the necessity for genetic encoding.

Different sensor classes offer distinct properties and advantages depending on intended use that cannot be fully explored here. Broyles *et al.*^38^ provide a comprehensive review of available dyes for optical mapping, while we have previously summarized dyes that are spectrally suitable for dual optical mapping and optogenetics^24^.

### Optical mapping hardware

Optical mapping systems integrate various sophisticated components to capture cardiac electrophysiology. *Figure 2* outlines key components, namely excitation sources, optical components, and high-speed camera(s). Common excitation sources include LEDs,^23^ tungsten–halogen lamps,^39^ mercury/xeon arc lamps,^27^ and lasers.^36^ Optical filters can narrow excitation wavelengths to avoid spectral cross-talk and effectively filter photons for imaging. In multi-parametric set-ups (see Multi-parametric imaging set-up section), further filters are employed to deconvolve the fluorescent signals. Optical lenses are used to focus excitation and emission photons.

Charge-coupled device (CCD)^23^ and complementary metal-oxide semiconductor (CMOS)^40^ cameras are most frequently used in optical mapping, and recent advances in CMOS technology have provided low-cost and portable imaging systems.^40,41^ Key camera metrics include quantum efficiency, dynamic range, signal-to-noise ratio, sampling rate, and pixel size. A high quantum efficiency, defined as the ratio of photogenerated electrons to incoming photons within each pixel, enables sufficient signal-to-noise ratio even with low fractional changes. More recently, back-illuminated CMOS cameras and electron multiplying charge-coupled devices (EMCCD) have been introduced to reduce noise and amplify emission signal, respectively, for increased sensitivity.^42^

A key consideration in optical mapping set-ups is the balance between spatial and temporal resolution. High temporal resolution (>500 Hz) is needed to capture millisecond scale changes in voltage and/or [Ca^2+^]_i_, while high spatial resolution (pixel size ≤ 100 s of microns) is required for detailed imaging of complex conduction patterns. Hardware constraints necessitate a trade-off between spatial and temporal resolution, where higher spatial resolution limits maximum sampling rate and vice versa.

More sophisticated optical mapping designs include panoramic, often multi-camera, all-optical stimulation and imaging, capturing the entire surface topology.^43^ Rieger *et al.*^44^ implemented a customized LED light source with 294 optical fibres for panoramic optical mapping of mouse hearts, expressing GEVIs. Importantly, panoramic view was captured through two lenses directing optical emission bands onto a single CMOS camera, thereby surpassing logistical complications of a multi-camera set-up.^45^

Probes with higher quantum yield, such as fluoVolt, require fewer photons for adequate signal quality and are compatible with two-photon microscopy techniques, capable of capturing tissue at greater depth.^29^ Multi-photon,^29^ optical coherence tomography^46^ and light sheet fluorescence microscopy techniques^47^ have allowed between 400 μm and 4 mm depth ranges, capturing transmural activation. Future advancements are required to optimize camera sensor quantum efficiency at near-infrared wavelengths for three-dimensional (3D) reconstruction (Z axis profiling) and transmural optical imaging.

### Data analysis

Short exposure times, small fluorescent changes, small pixel areas, and technical artefacts (e.g. motion and signal ‘blurring’ due to wavelength-dependent photon scattering^48^) all complicate processing and analysis of optical mapping data. Several approaches are applied to improve signal quality, including spatial and temporal filtering, temporal oversampling, and baseline correction. However, misapplication of these approaches (for example, by ‘over smoothing’ signals) can lead to misinterpretation, and the reader is directed to relevant literature that outline effective handling of optical mapping data.^35,49^

Recent advances have seen the development of several open-source options for optical mapping data analysis. These include general all-purpose software^49–51^ and more specialized options for arrhythmia analysis,^52,53^ panoramic imaging,^45^ conduction,^54^ and alternans.^55^ Further automation (e.g. machine learning–based approaches for automated artefact detection), combined with technical advances in minimizing post-processing needs, will further reduce the risk of misinterpretation of optical signals.

### Multi-parametric imaging set-up

Key physiological insights can be gained by combining voltage and Ca^2+^ imaging or other optically measurable parameters. Dual optical mapping is achieved by simultaneously exciting voltage and Ca^2+^ dyes with distinguishable emission wavelengths.^31^

Rh237 and rhod-2 are well suited for dual voltage and Ca^2+^ transient optical mapping set-ups, with similar peak excitation wavelength bands but distinct emission spectra.^24^ Furthermore, dual voltage and sarcoplasmic reticulum Ca^2+^ mapping can be achieved using rh237 and fluo-5N AM dyes.^56^ Dual mapping enables voltage–Ca^2+^ coupling analysis, including voltage–Ca^2+^ latency, important for elucidating arrhythmic risk.^57^

Triple-parametric optical mapping, recording voltage, Ca^2+^, and autofluorescent nicotinamide adenine dinucleotide (NADH), has been performed to investigate metabolism–excitation–contraction coupling.^58^ Optical mapping is also compatible with other imaging techniques. Caldwell *et al.*^59^ measured cAMP activity and voltage simultaneously using a combined optical mapping and Förster resonance energy transfer (FRET) set-up in murine whole hearts. Reactive oxygen species, oxygen, and mitochondrial membrane potential optical probes can provide additional metabolic insights.^60^

### Motion tracking

The heart is a dynamic organ. This presents a problem for optical mapping as motion can distort signal morphology.^61^ Therefore, cardiac optical mapping is usually carried out on non-beating hearts, omitting physiologically important bidirectional electromechanical feedback^62^ and altering metabolic demand.^63^ The pharmacological electromechanical uncoupler blebbistatin selectively inhibits myosin II isoforms, abolishing contraction.^64^ Blebbistatin has been reported to alter cardiac physiology, increasing action potential durations and Ca^2+^ transient upstroke rise times while reducing NADH autofluorescence in isolated murine hearts.^58^ However, the effect of blebbistatin on cardiac physiology is still disputed and contradictory findings may be due to species differences, blebbistatin concentration, or incorrect use (e.g. blebbistatin precipitation).^64,65^

Motion tracking by computational signal correction or ratiometry has made it possible to optically map the freely beating heart.^37,66^ Markers can be used to track and correct for movement.^67^ Motion correction can also be achieved without the use of fiducial markers, for example, by optical flow methods, which compute displacement vectors to quantify pixel movement and motion.^9,68^ Christoph & Luther *et al.*^68^ showed up to a 80% decrease in motion artefacts when using marker-free motion tracking in optical mapping videos of contracting hearts. However, two-dimensional (2D) motion tracking exclusively captures movements along the horizontal and vertical axes, omitting movements along the depth axis, thereby limiting accuracy. Zhang *et al.*^67^ performed 3D marker-based motion tracking to measure epicardial strain and deformation. Recent studies have shown how graphical processing units can be utilized to accelerate the application of open-source motion correction algorithms, demonstrating real-time correction of optical signals.^69^

Ratiometry requires two signals, generated either during excitation or emission,^36^ which are similarly distorted by motion but differentially respond to, for example, voltage or Ca^2+^. The effects of motion on the time series signal, and artefacts due to uneven dye loading and illumination, can therefore be eradicated. However, ratiometry alone does not ensure spatial coupling, so can only be used in preparations with minimal dispersion.^37^ There are also inherent limitations, including reduced effective frame rates. Several studies have combined motion tracking, ratiometry techniques and tracking images within each spectral band for further reduction in motion artefacts.^70,71^

Optical mapping of the freely beating heart is still a relatively specialized application; however, recent advancements will pave the way for innovative optical mapping investigations with enhanced physiological relevance.

## State-of-the-art applications of optical mapping

The advances outlined above have furthered the role of cardiac optical mapping as a central research tool, facilitating several important insights in cardiac electrophysiology and arrhythmogenesis, for example, the genetic basis for atrial fibrillation,^72^ and insights into the chamber specificity of anti-arrhythmics.^73,74^

Human iPSC-CMs are an increasingly popular model for cardiac research and drug screening, capable of modelling patient-specific cell lines. The field is moving towards organoid EHTs, often utilizing human iPSC-CMs, to mimic cell–cell interactions for higher physiological relevance. However, chemical Ca^2+^ dyes have been demonstrated to significantly impair blebbistatin efficacy in cardiac single-cell models, including human iPSC-CMs, increasing risk of motion artefacts.^75^ While microinjection has been suggested to reduce intracellular dye concentrations and subsequent adverse effects, this is not scalable.^76^ Mapping of beating human iPSC-CMs and EHTs using computational motion tracking has provided a solution, additionally capturing Ca^2+^–contraction coupling, for accurate mapping of these models.^9^

The advent of both red-shifted dyes (e.g. di-4-ANBDQBS) and higher quantum yield dyes (e.g. fluoVolt) has increased single wavelength penetration depths from 0.5–1 mm using standard (green–red) dye to ∼1–4 mm in cardiac tissue using ‘near-infrared optical mapping’.^29,77^ Previously, studies used cardiac wedge preparations or myocardial slices to provide a 2D transmural surface^10,57^ or two CCD cameras on either side of the myocardium in ventricular wall preparations.^78^ Mitrea *et al.*^79^ applied near-infrared di-4-ANBDQBS for improved transillumination to record signals from four different layers of the myocardial wall. Furthermore, longer wavelengths of near-infrared dyes demonstrate reduced absorption by blood, offering opportunity for *in vivo* cardiac optical mapping.^27^ Hansen *et al.*^80^ performed the first *in vivo* cardiac optical mapping using near-infrared di-4-ANBDQBS dye for successful activation mapping of the canine left atrium during sinus rhythm and fibrillation. More recent studies have used excitation ratiometry or mechanical stabilization to reduce motion artefacts during *in vivo* cardiac optical mapping^23,81^; however, neither approach was sufficient for broad-area, accurate APD measurement. Advances in motion tracking algorithms, combined with red-shifted dyes and novel signal processing, have improved optical mapping capabilities for *in vivo* preparations.^82^ However, challenges remain including invasive surgery, limited optical view determined by the surgical thoracic window, and adverse effects of anaesthesia on cardiac electrophysiology.

Multi-parametric optical imaging unveils novel sequences of events in excitation–contraction coupling in cardiac disease. Hypertrophic murine hearts demonstrated prolonged voltage–Ca^2+^ latency, indicating altered electrical–contraction coupling.^57^ Dual optical mapping also captures cross-talk between electrical and Ca^2+^ alternans, revealing positive or negative electromechanical coupling.^83^ Voltage and sarcoplasmic reticulum Ca^2+^ signals simultaneously recorded in isolated rabbit hearts during ventricular fibrillation indicated ryanodine receptor refractoriness triggered sarcoplasmic reticulum Ca^2+^ alternans, subsequently leading to electrical alternans.^56^

Triple-parametric optical mapping using Ca^2+^-sensitive rhod-2-AM, voltage-sensitive rh237, and endogenous NADH fluorophores revealed metabolic changes precede cardiac electrophysiology changes in murine ischaemic hearts.^58^ Recently, Caldwell *et al.*^59^ measured cAMP and electrical activity simultaneously, reporting higher apical phosphodiesterase activity in females vs. males which may contribute to sex-dependent electrophysiological variation.

### Mechanistic understanding from optical mapping

Mapping of the freely beating heart has enabled excitation–contraction coupling and electromechanical feedback to be captured. Christoph *et al.*^84^ studied the spatiotemporal dynamics and topological organization of electrical and mechanical rotors in sinus rhythm and ventricular fibrillation. Three-dimensional mechanical scroll waves of contracting pig hearts were captured using combined panoramic optical mapping and four-dimensional (4D) ultrasound. Marker-free motion tracking for simultaneous optical mapping of voltage, [Ca^2+^]_i_, and mechanical strain demonstrated cardiac tissue deformation was related to onset of electrical activation.^84^ Additionally, optical mapping of isolated, contracting pig hearts using excitation ratiometry demonstrated electromechanical decoupling during atrial fibrillation and highlighted Ca^2+^ remodelling as an important mediator in early stages of atrial fibrillation.^85^

Combining optical mapping with multi-modal 3D structural analysis has facilitated significant advances in mechanistic understanding of pathophysiological conduction and arrhythmia. Optical voltage mapping and tissue clearing approaches have revealed neurocardiac and myofibre remodelling post-infarct unique to the border zone region, which contributes to heterogeneous conduction and therefore arrhythmia risk.^86^ Transmural near-infrared optical mapping has been combined with MRI, for improved clinical detection of re-entrant atrial fibrillation drivers.^77^ Optical action potentials were recorded over a depth of ∼4 mm to capture intramural microanatomic re-entry, commonly misinterpreted as focal patterns by ‘surface’ multi-electrode mapping. Combination with MRI assessment of intramural fibrosis enables delineation between re-entrant AF drivers and non-drivers, important for improving the efficacy of driver-targeted ablation.

Optical recording of the transmembrane voltage has the unique advantage of not being corrupted by signal artefacts during electrical stimulation, for example, during defibrillation. Therefore, optical mapping has been fundamental in investigating the virtual electrode phenomenon, whereby an external stimulus polarizes the cardiac tissue, generating positive and negative virtual electrodes.^14^ Virtual electrode theory can be harnessed to better understand mechanisms of cardiac defibrillation, where the interaction between the shock-induced virtual electrodes and the ongoing electrical activity can terminate re-entrant circuits or, conversely, reinduce arrhythmia.^14^ Efimov *et al.* demonstrated optimal biphasic shocks achieved successful defibrillation in rabbit hearts without arrhythmia reinduction. Designing effective shock waveforms that capitalize on virtual electrode effects may improve defibrillation success and minimize potential side effects.

## Principles of optogenetics

**Figure 3:**
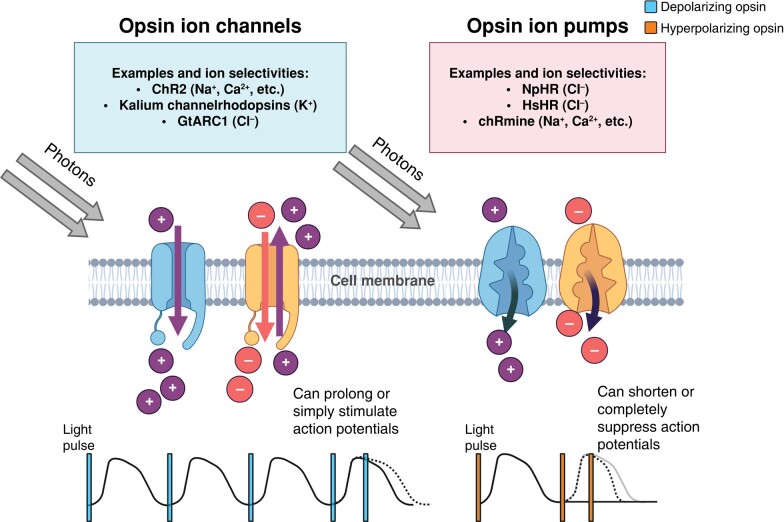
Mechanisms of depolarizing and hyperpolarizing opsins in manipulating cardiac electrophysiology and examples. Upon photostimulation, depolarizing opsins enable cellular cation influx and hyperpolarizing opsins enable cellular anion influx or cation efflux. Light-activated depolarizing opsins generate optically stimulated action potentials or prolonged action potentials if light pulse is delivered during repolarization. Light-activated hyperpolarizing opsins completely inhibit action potentials or shorten action potentials if light pulse is delivered during repolarization. ChR, channelrhodopsin; NpHR, *Natronomonas pharaonic* halorhodopsin; HsHR, *Halobacterium salinarum* halorhodopsin. Figure created on BioRender.com.

Optogenetics, initially developed to study complex neuron interactions, has since been translated to cardiac applications with great effect.^17^ On wavelength-dependent illumination, opsins enable ionic transport across the cell membrane in a similar manner to voltage-gated, ligand-gated, or mechano-sensitive ion channels and pumps (*Figure 3*). Optogenetics has made possible several mechanistic insights and may potentially advance to clinical application.

### Opsin design and mechanism

#### Channelrhodopsins

The most popular family of opsins are channelrhodopsins (ChR). ChR2 is the most common of these, a low-selectivity cation channel with high photosensitivity at ∼480 nm (blue) excitation wavelengths enabling passive ion movement for dynamic stimulation and depolarization.^87^ Several studies have used ChR2 for pacing cardiac preparations.^18,88,89^

Since the first deployment of ChR2, several mutations have been genetically engineered. Chen *et al.* designed and validated ChRmine, a red-shifted excitation wavelength opsin, for non-invasive, *in vivo* optogenetic cardiac pacing in mice.^90^ Lin *et al.*^91^ engineered red-shifted opsin ReaChR with reduced tissue absorption and scattering, resulting in greater photocurrents. Additionally, other opsin variants with greater photocurrent conductance have been published, such as ChR2-H134R, ChR2-T159C and ChR-XXL, and CatCh.^24^

ChR2 containing a mitochondria-targeting sequence at its N-terminus has been shown to successfully reach the inner mitochondrial membrane in neonatal rat cardiomyocyte cells, to optically control mitochondrial membrane potential and ATP synthesis.^92^ Additionally, sensor co-expression has enabled dual functionality of optical stimulation and transduction signalling recording using FRET.^93^

#### Halorhodopsins, anion, and Kalium channelrhodopsins

Conversely, opsins such as halorhodopsins drive anion transport.^94^ Halorhodopsins *Natronomonas pharaonic* halorhodopsin (NpHR) and *Halobacterium salinarum* halorhodopsin (HsHR) exhibit extracellular chloride affinity and pump chloride ions (Cl^−^) into the cell upon light stimulation causing hyperpolarization.^94^ Anion ChRs such as GtARC1 enable Cl^−^ conductance and can be used to depolarize cardiomyocytes^95^ or, through sustained depolarization, block re-excitation and inhibit cardiac action potentials,^96^ although this may be proarrhythmic due to sodium (Na^+^)/Ca^2+^ overload. HcKCR1, a natural Kalium ChR (KCR), was recently discovered as the first ChR which selectively conducts potassium (K^+^) over Na^+^ ions, rapidly hyperpolarizing (<1 ms response time) murine cortical neurons.^97^ WiChR, another KCR, was recently expressed in human iPSC atrial cardiomyocytes and inhibited action potentials, reversibly suppressing spontaneous contraction during blue light illumination.^8^ Spectrally distinct depolarizing and hyperpolarizing opsins can be expressed and actuated in tandem, to enable bidirectional optical modulation.^94^

#### Limitations of opsins

Cytotoxicity can be a concentration-dependent side effect of opsins.^98^ Additionally, after repetitive stimulation, several ChR variants can demonstrate desensitization, including reduced peak and plateau currents, thereby reducing opsin efficacy.^99^ Interval switching protocols^100^ or complete dark adaption of ChRs^99^ can prevent desensitization or eliminate bias, although may alter photocurrent. Further optimization of opsin safety, efficacy, and durability is required to expand utility of optogenetics, including potential clinical applications.

### Opsin delivery

Key to the application of optogenetics is the expression of opsins by target cells/tissues. Model species, targeted cell type, vector genome size, duration of expression, and expression level required must all be considered for opsin delivery efficiency.^98^ The main methods of opsin delivery are summarized in *Table 1*.

**Table 1:** Summary of main opsin delivery methods and characteristics

Opsin delivery method	Vector	Cell-targeting method	Characteristics	References
Viral transduction	Adeno-associated virus (AAV)	Pseudotyping (glycoproteins, antibodies) or cell-specific promoters	- No host genome integration - Suitable for post-mitotic cells (e.g. cardiomyocytes or neurons) - Smaller packaging capability (<4.7 kb) - Self-complementary AAVs show improved transduction speed - High durability and stability of expression with low immunogenicity	^90,99,101–105^
	Lentiviral		- Host genome integration - Suitable for mitotic and stem cells - Larger packaging capability (8–10 kb) - Slower transduction speed/diffusion	
Electroporation	Opsin-encoding plasmid	Cell-specific promoters	- Fast process - Versatile (suitable for most cell types) - Loss of cell viability/toxicity - Non-targeted transfection - Safety concerns for *in vivo* gene delivery	^106^
Lipid-based transfection	Opsin-encoding plasmid	Cell-specific promoters	- Versatile - Lower transfection efficiency vs. viral - Non-targeted transfection	^107^
Tandem-cell unit	Non-excitable opsin-expressing cells	Non-excitable opsin-expressing (donor) cells couple to target cells via gap junctional proteins for indirect photostimulation	- Improved safety compared with viral methods - Energy-efficient - Relies on cell coupling which may be affected by pathology - Requires introduction of donor cells, presenting *in vivo* challenges	^108^
Carbon and gold nanoparticles	Genetic construct	Nanoparticles carry ligands for cell-specific binding	- High efficacy, selectivity, and speed - Multi-functional - Sustained release and opsin expression - Potential for *in vivo* application - Complex	^109,110^
Transgenic animal models	Genetic construct	Opsin gene introduced during specific time point of embryonic development	- Commonly used in mice models - Achieves specific, stable opsin expression - Less compatible with non-human primates - High maintenance costs, long generation cycles, and ethical concerns	^22,111^
	Genetic construct	Cre-Lox system		

The model and method of opsin delivery must be compatible. For example, lentivirus undergoes host genome integration unlike adeno-associated virus, providing a compatible vector for mitotic and stem cell optogenetic models.^101^ The most widely used method is viral transduction using adeno-associated virus (AAV), offering rapid and consistent transduction with cell specificity (viral tropism).^99,102^ Cardiac troponin T is a commonly selected promoter for cardiomyocyte-targeted opsin expression.^90^ Adeno-associated virus vectors display longer, more stable expression of opsins and minimal inflammatory response in post-mitotic cells, such as neurons or cardiomyocytes, compared with lentivirus.^103–105^ Self-complementary AAV vectors have recently been designed, eradicating the DNA synthesis step which AAVs normally undergo, increasing viral transduction speed.^103^ While non-viral opsin delivery methods including physical (electroporation) and chemical (liposomes, tandem-cell unit, nanoparticles, and transgenic models) approaches usually allow high stability of opsin expression, they demonstrate lower specificity and/or poorer clinical translatability.

### Illumination

Direction and focus of light aids region-specific photostimulation, alongside genetic targeting. Most opsins are activated by shorter, ‘blue’ wavelengths, associated with reduced tissue penetration, providing only surface actuation.^105^ Therefore, transmural conductance and dyssynchrony cannot be optically modulated, despite established importance in arrhythmogenesis.^112,113^ Additionally, studies have demonstrated the significance of depth of photostimulation to terminate re-entrant arrhythmias.^114,115^ Strategies with deeper penetration capabilities have been explored, including ultra-thin injectable optoelectronic devices, flexible biocompatible membranes, and integration of opsins with X-ray excitable nanophosphors or upconversion nanoparticles.^116,117^ Ultra-thin, waterproof micro-LED arrays on flexible substrate can be implanted *in vivo* to deliver cardiac illumination for transmural optical stimulation.^117^ Near-infrared wavelengths allow significantly greater tissue penetration and reduced light-induced cytotoxicity.^118^ Upconversion nanoparticles can transduce low-energy near-infrared photons to high-energy infrared, visible, or ultraviolet photons.^119^ Yu *et al.*^102^ demonstrated addition of all-trans-retinal (ATR) photosensitizer to ChR2-H134R-expressing cardiomyocytes *in vitro* could significantly increase ChR2 membrane expression and reduce optical pacing energy. However, techniques altering opsin spectral sensitivity and ATR treatment can compromise opsin kinetics and cardiac electrophysiology respectively.^102^

Unlike optical mapping where homogeneous illumination is preferred, optogenetics often employs directed illumination signals. Liquid crystal and digital micromirror devices, incorporating an array of microscopic mirrors, can acutely control LED-derived light impulses to narrow focus and increase spatial targeting.^120^ Moreover, light impulse patterns in optogenetics have shown to be important in arrhythmia termination studies.^121^

Standard optical sources, such as LEDs, are bulky and therefore must be positioned externally to any *in vivo* preparations. Multi-LED probes in the form of LED-chips have been implanted into the septum of *ex vivo* mouse hearts, expressing ChR2, and enabled stable optogenetic pacing including endocardial actuation.^88^ While this still requires a complex surgery and risk of complications, these set-ups provide higher clinical translatability.

## Applications of optogenetics in cardiac electrophysiology

Optogenetics has revolutionized cardiovascular research, with Bruegmann *et al.*^18^ first demonstrating its application for *in vivo* murine heart pacing in 2010. Subsequently, continued advances have broadened optogenetics application to several domains. For example, red-shifted opsins (e.g. ChRmine) have enabled non-invasive, *in vivo* optogenetic cardiac pacing in freely moving mice wearing micro-LEDs.^90^ Implantable multi-LED devices delivering apical or transthoracic illumination with a closed chest have enabled *in vivo* arrhythmia termination in pathologically remodelled rat hearts, expressing ReaChR.^122,123^

Patterned illumination techniques have been employed for precise regional stimulation of the heart. For example, Arrenberg *et al.*^124^ located and stimulated zebra fish cardiac pacemaker cells through altering light patterns and selective plane illumination. Additionally, acute photostimulation has been employed to dynamically alter action potential duration, ranging from depolarizing opsins at precise phases of the action potential to eliminate arrhythmia^125^ to complete silencing of action potential firing.^126^

Targeted delivery methods facilitating cell-specific opsin expression have paved the way for comprehensive electrophysiological characterization and studying interactions between different cardiac cell types. Zaglia *et al.*^22^ crossed double-floxed ChR2-tdTomato mice with connexin-40-Cre mice to induce cardiac conduction system-specific opsin expression and reported myocardial ectopy sites correlated with Purkinje fibre connection. Nussinovitch *et al.*^89^ demonstrated the ability of ChR2-expressing mouse embryonic fibroblasts to pace neonatal rat-derived cardiomyocytes in co-culture, in response to blue light flashes.

Optogenetics has also offered a unique approach to study the cardiac autonomic nervous system. For instance, Moreno *et al.*^127^ demonstrated heart rate reduction via stimulation of ChR2-expressing intrinsic parasympathetic neurons in mouse hearts. This illustrates the potential for optogenetics in mimicking complex pathophysiology underlying cardiac disease.

In an *in vivo* study, Rao *et al.*^128^ successfully paced rat hearts using upconversion nanoparticles embedded in flexible polydimethylsiloxane films attached to the ventricle. This approach effectively modified near-infrared light spectra for ChR2 activation. Furthermore, pacing efficiencies were comparable with blue light pulsed stimulation, presenting a promising non-invasive method of cardiac rhythm modulation.

Finally, optogenetics plays a critical role in inducing, studying, and terminating arrhythmias.^20,129–132^ For instance, Lemme *et al.*^100^ used optogenetic methods to pace 3D EHTs derived from human iPSC-CMs, simulating conditions like chronic tachypacing-induced cardiac dysfunction. Chronic optogenetic tachypacing correlated with greater vulnerability of EHTs to electrical burst pacing-induced tachycardia, as well as reduced action potential duration and effective refractory period.^100^ Further, optogenetic defibrillation with continuous blue light was successful in terminating arrhythmias induced by burst pacing.

## Cardiac all-optical electrophysiology

The application of cardiac all-optical imaging, combining optogenetics and optical mapping for acute control and sensing of cardiac electrophysiology, is expanding.^41^ Cells expressing opsins in conjunction with fluorescent dyes, or genetically encoded indicators for all-genetic delivery,^133^ enable contactless, cell-selective all-optical control and sensing. For an all-optical set-up, compatible opsins and sensors are essential, ensuring minimal spectral overlap.^24^

All-optical set-ups have been used for pacing and imaging human iPSC-CMs,^40,134^ cardiomyocyte subpopulations,^135^ ventricular slices,^115^ and *ex vivo* hearts.^22^ Red-shifted dyes facilitate all-optical electrophysiology as they are spectrally distinct from ChR2. Optical mapping, using di-4-ANBDQBS, of optically paced ChR2-expressing rat hearts demonstrated reduced total ventricular activation time and improved homogeneity of ventricular depolarization vs. electrical pacing.^136^ These findings suggest that broad-area optical stimulation may offer an improved method for cardioversion therapy in patients with mechanical cardiac dyssynchrony.

## Translational applications of cardiac optical methods

Although still far from clinical application, optogenetic pacing and defibrillation show promising translatability.^125^ The current gold standard for restoring sinus rhythm is defibrillation by electrical cardioversion, which is painful and not cell selective. Whilst effective, standard clinical pacemaker devices require complex surgery. One study reported 9.5% of cases show post-surgery complications within 6 months, such as pacing lead dislodgement and infection.^137^ Consequently, there has been a large research effort exploring optogenetic cell-selective and pain-free arrhythmia termination.

### Optoelectronic devices for cardiac pacing and defibrillation

**Figure 4:**
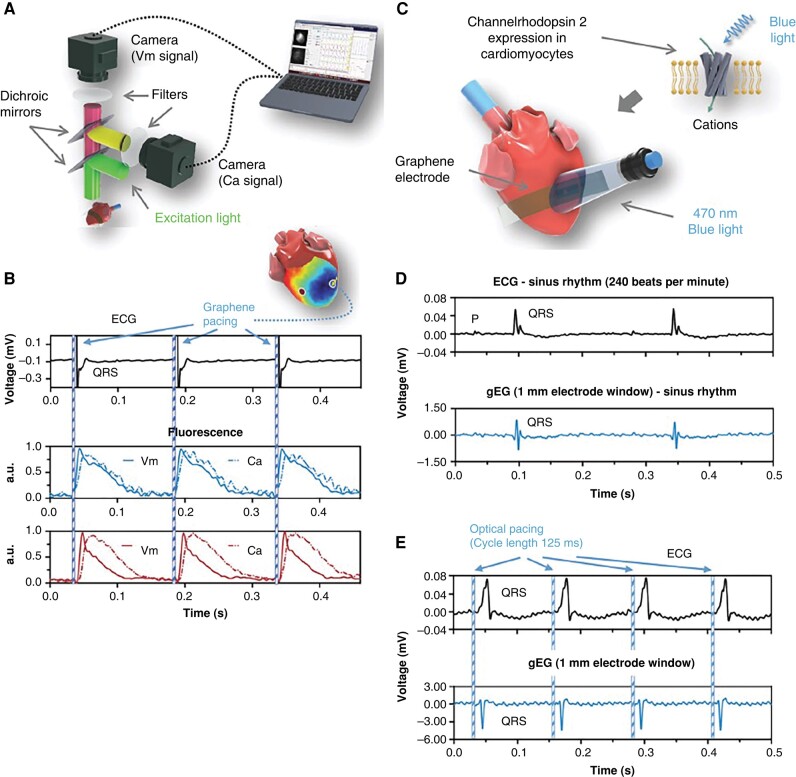
Previously published optoelectronic device, encompassing a flexible graphene array biointerface compatible with optical sensing and actuation. (*A*) Schematic of graphene device compatible with dual optical mapping [voltage (Vm) and calcium (Ca^2+^)]. (*B*) Traditional electrocardiogram (ECG) and fluorescent voltage (rh237) and Ca^2+^ (rhod-2-AM) signals acquired by dual optical mapping of *ex vivo* mouse hearts during pacing by unipolar, transparent graphene electrode. (*C*) Schematic of graphene array device compatible with optogenetics in *ex vivo* mouse hearts with channelrhodopsin 2 (ChR2) cardiomyocyte-specific expression. (*D*) Traditional ECG and graphene electrogram recordings during sinus rhythm. (*E*) Traditional ECG and graphene electrogram recordings during optogenetic pacing at 125 ms cycle length. Figure reprinted and adapted with permission from^139^ by Lin et al.

Optoelectronic devices provide opportunity for accurate, real-time modulation of cardiac electrical activity for *ex vivo* or *in vivo* set-ups, offering exciting research and future clinical applications. Miniaturization of optoelectronic devices has enabled previously unattainable *in vivo* application in rodents.^138^ Implanted, bioresorbable optoelectronic devices have achieved *ex vivo* concurrent cardiac pacing and electrophysiology measurement^139,140^ (*Figure 4*) and *in vivo* acute and chronic cardiac pacing.^138,140^

Recently engineered, transparent graphene electrode arrays were compatible with *ex vivo* cardiac optical sensing and stimulation, in addition to generating electrograms.^139,141^ Xu et al.^142^ developed 3D elastic membranes to envelop the rabbit heart with flexible arrays overlaid, consisting of micro-LEDs for optical pacing and multi-functional sensors for pH, temperature, and strain measurement.

Closed-loop all-optical pacing and modulation has been performed in real time (≤2 ms response time) to control electrical activity in *ex vivo* mouse hearts and restore sinus rhythm in abnormal conditions.^120^ Multi-functional optoelectronic devices may offer advanced cardiac diagnostic and treatment applications; however, feasibility of a closed-loop, implantable clinical pacemaker device may be limited due to the amount of spatiotemporal data to be processed in real time. These limitations however may be overcome by advancements in ‘on-chip’ computing technologies.

Nevertheless, closed-loop optogenetics provides a promising research tool for previously unverifiable hypotheses, such as determining the level of discordance in alternans required to trigger arrhythmia.^120^

### Pre-clinical cardiotoxicity drug screening

All-optical interrogation could offer an improved pre-clinical cardiotoxicity screening platform for novel drugs. The current gold standard is electrode-based or patch-clamp recording to measure QT-prolongation and human ether-a-go-go-related gene channel inhibition, which provides limited information on conduction or contractility defects.^143^ A multi-parametric, all-optical set-up would enable multiple facets of cardiac physiology to be tested, including metabolism–excitation–contraction coupling, for more accurate cardiotoxicity predictions.^58,134,144^

### Challenges for clinical application of optical methods

A key barrier to clinical optogenetics is the safety and efficacy of opsin delivery and expression. Ongoing clinical trials are assessing the long-term safety of gene therapy to induce opsin expression,^145^ marking a crucial step in advancing this field.

A considerable limitation of optogenetic-based therapy is light attenuation due to photon scattering and absorption, causing transmural gradients and potentially arrhythmia. Thus, implementing systems and opsins that allow greater penetration depth at safe irradiances must be prioritized. Advancing optoelectronic devices for clinical pacing and defibrillation also requires optimizing illumination strategies for successful and energy-efficient defibrillation of different heart rhythm pathologies using pre-clinical models. Device sensitivity for arrhythmia detection should be improved using artificial intelligence (AI) approaches, although developing and validating algorithms will require a large training data set.^146^ The long-term effects of optogenetic pacing on cardiac electrophysiology and structural remodelling must be investigated. Additionally, future studies should confirm whether optical pacemakers require less energy since they only target a subgroup of cells, which may improve longevity over standard clinical pacemakers.

Combined near-infrared optical mapping and 3D functional imaging provides promising potential for improving arrhythmic driver-targeted catheter-based ablation^77^. However, this technique requires further *in vivo* study before progressing to clinic. Additionally, while pre-clinical *in vivo* optical mapping studies have been performed successfully, inherent limitations including field of view, invasive surgery, and penetration depth remain. Similarly, although all-optical imaging provides a contactless, high-resolution research method for precise cardiac electrophysiology actuation and measurement, the logistical limitations of optical mapping imaging systems restrict clinical translatability.

## Utilizing computational modelling and artificial intelligence in optical imaging and control

Advanced computational approaches have been paramount in optical electrophysiology and are increasingly important for clinical translation. Deep learning predictive modelling has been implemented to compute phase maps and phase singularities in real-time using brief temporal sequences of electrical activity from optical maps,^147^ which may enable more accurate analysis of rotors and arrhythmia. Additionally, these models can predict future phase maps and phase singularity positions, targeting the ‘excitable gap’ as a low-energy method for arrhythmia termination.

Computational optogenetic models have also provided insight into spatial targeting for successful arrhythmia termination. Models revealed light pulses should last longer than the arrhythmia cycle length^148^ or provide precise optogenetic spatial–temporal control to drag rotors to non-excitable boundaries^149^ to reliably terminate arrhythmia. Computational optogenetics has also been applied and validated to study opsin kinetics and dynamics, across a range of voltage and irradiance conditions,^150^ potentially accelerating the development of clinically suitable candidates.

## Conclusions

Optical mapping and optogenetics have undoubtedly revolutionized the field of cardiac electrophysiology research, offering unique insights into cell–cell interactions, arrhythmia development, and defibrillation strategies. All-optical imaging has facilitated contactless cardiac electrophysiology investigation, and implantable optoelectronic cardiac pacemakers could enable pain-free, cell-selective defibrillation. Advances in optical imaging technologies and tools, to improve penetration depth, alongside long-term safety studies will further enhance clinical applications of optical imaging.

## Data Availability

Not applicable.
